# The Development of a Veterans Health Administration Emergency Management Research Agenda

**DOI:** 10.1371/currents.dis.c0c84b1b680388649227be71823e6adf

**Published:** 2017-03-23

**Authors:** Aram Dobalian, Maria Claver, Deborah Riopelle, Tamar Wyte-Lake, Ismelda Canelo

**Affiliations:** Veterans Health AdministrationDepartment of Veterans Affairs; Veterans Emergency Management Evaluation Center and VAGLAHS HSR&D Center of Excellence for the Study of Healthcare Provider Behavior, California State University, Sepulveda, California, USA; Veterans Health Administration, HSR&D Center for the Study of Healthcare Innovation, Implementation & Policy (CSHIIP), Los Angeles, California, United States; Veterans Administration, Veterans Emergency Management Evaluation Center, North Hills, California, United States; VA Greater Los Angeles Healthcare System, VA HSR&D Center for the Study of Healthcare Innovation, Implementation and Policy (CSHIIP), North Hills, California, United States

## Abstract

**Introduction:**

The Veterans Health Administration (VHA), the largest integrated healthcare delivery system in the United States, is charged with ensuring timely access to high-quality care for veterans during disasters, and supporting national, state, local, and tribal emergency management and homeland security efforts. In 2008, the VHA Office of Public Health (OPH) sponsored the first VHA Emergency Management Research Agenda-setting conference to develop research priorities that address the needs of veterans and to position VHA as a national leader in emergency management by having VHA serve as a “laboratory” for the development of evidence-based emergency management practices.

**Methods:**

We focused on four steps: #1: Appraising the emergency management research portfolio of VHA-based researchers; #2: Obtaining systematic information on VHA’s role in emergency management and the healthcare needs of veterans during disasters; #3: Based upon gaps between the current research portfolio and the existing evidence base, identifying strategic priorities using a research agenda-setting conference; and #4: Laying the groundwork to foster the conduct of emergency management research within VHA.

**Results:**

Identified research priorities included how to prevent and treat behavioral health problems related to a disaster, the efficacy of training programs, crisis communication strategies, workforce resilience, and evacuating veterans from health care facilities.

Conclusion: VHA is uniquely situated to answer research questions that cannot be readily addressed in other settings. VHA should partner with other governmental and private entities to build on existing work and establish shared research priorities.

## Introduction

In addition to providing healthcare for veterans, the United States Veterans Health Administration (VHA), part of the Department of Veterans Affairs (VA), is charged with improving the Nation’s preparedness for response to war, terrorism, national emergencies, and natural disasters by developing plans and taking actions to ensure continued service to veterans, as well as supporting national, state, and local emergency management, public health, safety and homeland security efforts.^1^^,^^2^^,^^3^ Under the National Response Framework of the United States (U.S.), VHA has support responsibility under 7 of the 15 Emergency Support Functions including emergency management, public health, and medical services. Consequently, VHA has directly provided care for veterans—and sometimes non-veterans—during every major national disaster since 1992.

By law, VHA provides health care to enrolled Veterans first, but can provide support to communities when there are local emergency needs or federally declared disasters. In this capacity, VA’s extensive resources as the largest integrated healthcare delivery system in the U.S. (more than 350,000 personnel at more than 1,300 medical sites serving about 9 million Veterans) may be used to support other Federal and state agencies and local communities by providing public health and medical services following emergencies and disasters. For example, VA deployed more than 1,300 personnel and 12 mobile clinics to Louisiana and Mississippi following Hurricane Katrina; the clinics provided care to about 15,000 patients, including 11,000 who were not Veterans. The VA also provide significant amounts of care to individuals impacted by events as diverse as the World Trade Center attacks on September 11, 2001,^4^ and the earthquake in Haiti that occurred in 2010. VA staff regularly participate in emergency planning for events such as domestic Olympics, Presidential Inaugurations, and Papal visits.

In mid-2008, VHA’s Office of Public Health (OPH, now part of the VHA Office of Patient Care Services) provided funding to researchers at the Health Services Research & Development (HSR&D) Center of Excellence for the Study of Healthcare Provider Behavior to develop a plan to establish a comprehensive, VHA emergency management research and program evaluation agenda. It was hoped that the agenda would provide a basis for fostering the conduct of more VHA-based emergency management research, and, over time, position VHA as a national leader in emergency management research. This paper summarizes the process and outcomes of this effort, and outlines VHA’s research and evaluation priorities.

## VHA Emergency Management Research Agenda-Setting Process

Emergency management professionals and researchers with significant funded or published research on emergency preparedness were invited to join a conference wherein attendees would assist in the creation of a research agenda designed to address gaps in research on VHA emergency management. The planning group operated within the framework of a four-step action plan adapted from Yano and colleagues (2006)^5^ for a VHA Women’s Health Research Agenda-Setting Conference (Table 1).

**Table 1. Four step action plan toward a VHA emergency management research agenda:** 

Action Plan	Approach
Step #1: Critically appraise the VHA emergency management research portfolio	1. Obtain and review history of emergency management funding to VHA researchers; 2. Analyze data by funding source (e.g., VHA, other federal, private, foundation)
Step #2: Obtain systematic information about VHA emergency management to provide an evidence base for the research agenda	Conduct a systematic VHA emergency management literature review, including review of grey literature as well as peer-reviewed literature
Step #3: Based on gaps between the current VHA research portfolio (step #1) and the assessment of the evidence base (step #2), identify priorities for a VHA emergency management research agenda	1. Review and adapt priority-setting strategies by other agencies (e.g., Department of Defense, Centers for Disease Control and Prevention); 2. Review VHA strategic plans3. Conduct gap analysis, priority setting, and consensus development during a VHA emergency-management research agenda setting conference (held July 2009)
Step #4: Foster the conduct of VHA emergency management research	1. Build research capacity through collaboration, networking, and mentoring; 2. Increase awareness and visibility of VHA emergency management research

**Appraisal of VHA’s Research Portfolio**

To support strategic planning, it was necessary to assess the current state of existing emergency management research within VHA. This historical research portfolio provides a foundation for the reader to understand both the nature and scope of the then current VA emergency management research portfolio upon which the conference planning and subsequent research agenda were based. The following section reviews emergency management research funding secured by VHA researchers through 2008.

*Emergency Management Funding*

During 2003-2008, VHA researchers based at one of the HSR&D Centers of Excellence were funded by HSR&D and the VHA Office of Mental Health Services, and non-VHA sources, including the Agency for Healthcare Research and Quality (AHRQ), the Centers for Disease Control and Prevention (CDC), and the National Institutes of Health (NIH) (Table 2).

**Table 2. VHA and non-VHA funding for emergency management research:** 

Fiscal Year	VHA	Non-VHA	Total
2003	$354,279	$131,500	$485,764
2004	$441,336	$851,859	$1,293,195
2005	$489,235	$466,395	$955,630
2006	$523,910	$958,709	$1,482,619
2007	$385,896	$598,982	$984,878
2008	$0	$1,370,472	$1,370,472
	Total Number of Studies: 6	Total Number of Studies: 11	

VHA-funded emergency management research increased steadily from 2003 to 2006, then decreased by more than 25% in 2007. No VHA researchers were funded by VHA to conduct emergency management research during 2008. Non-VHA funding increased more than sixfold from 2003 to 2004, then decreased by about half the following year. From 2006 to 2008, non-VHA funding to VHA researchers who study emergency management continued to fluctuate. Some of the fluctuation in funding from year to year during this time period may be due to the cyclical nature of funding related to the occurrence of major disasters. For example, it is likely that the increase in funding for emergency management research in 2005 and 2006 was a response to Hurricanes Katrina and Rita.

It is evident from this review that emergency management is an emerging area of research in terms of VHA funding. As shown in Table 3, emergency management funding has remained relatively constant at under one percent of total funding to VHA researchers based at HSR&D Centers of Excellence. In comparison, former emerging VHA research topics such as women veterans’ health started at approximately 2-3% of total funding to VHA researchers.^5^ Subsequent to the VHA Women’s Health Research Agenda-Setting Conference in 2004, women’s health was identified as a VHA research priority, leading to an increase in funding for women veterans’ health research.

**Table 3. Emergency management research as a percentage of total VHA and non-VHA funding to VHA-based researchers:** 

	Emergency management research funding	Total research funding	% of Total
2005	$955,630	$160,750,113	0.6%
2006	$1,482,619	$156,282,076	0.9%
2007	$984,878	$165,994,469	0.6%
2008	$1,370,472	$170,103,121	0.8%

In summary, the total amount of emergency management research funding secured by VHA researchers stayed relatively constant from FY 2004 to FY 2008, although VHA funding declined to zero in FY 2008. Non-VHA funding decreased in FY 2005, but increased again beginning in FY 2006. VHA funding comprised 34%-73% of the total funding VHA emergency management researchers secured from FY 2003 to FY 2007. NIH was the primary sponsor of this type of research during FY 2007 and FY 2008.

The number of VHA-based researchers funded by VHA sources increased from 9 during FY 2003 to a high of 27 in FY 2007, before declining to zero during FY 2008. In contrast, the number of VHA-based researchers funded by non-VHA sources increased from 1 during FY 2003 to a high of 6 during FY 2007, before dropping to 3 during FY 2008.

The decline in VHA funding in FY 2008 in part reflects the small number of studies in progress during this time period, and suggests a need for more consistent funding in this area in order to attract and grow the emergency management research community within VHA. These trends suggest a clear interest among the VHA community for conducting research in the area of emergency management, but one that is limited by cyclical factors such as variations in funding over time.

**Establishing the Evidence Base for Agenda Development**

*Research Portfolio*

A review of HSR&D-funded studies provides a broad overview of emergency management issues confronting VHA. A systematic search within VHA databases identified six HSR&D-funded studies since 2002. One of the six studies focused on surveillance, two on education, and three on the response to bioterrorism or natural disasters (two of which focused on vulnerable populations).

*Summary of HSR&D Studies*

The six HSR&D-funded studies since 2002 are limited in scale and scope (Table 4). The one surveillance study points to the importance and usefulness of automated monitoring of electronic health information in early illness detection. The two educational studies, involving providers and patients, indicate an existing need to adapt educational interventions to the VHA population. Of the three response studies, two evaluated actual events and one involved scenario-modeling. Collectively, they addressed VHA’s ability to compare responses across locations and time, the ability to study vulnerable populations, and the recognition of VHA as a potential target due its governmental affiliation.

**Table 4. Summary of VHA HSR&D Service-funded studies on emergency management:** 

PI	Background	Objective	Methods	Findings
				
S. Delisle(IIR 06-119)	Early detection is critical for infectious disease outbreaks of public health importance. Disease surveillance can be potentially enhanced through automated monitoring of electronic medical records compared to manual case reporting systems	To automate the use of data from VHA’s computerized patient record system (CPRS) to enhance outbreak detection by including illness progression and severity to reduce “noise” of common syndromes	Clinical data was grouped by respiratory disease severity using diagnostic and procedures notes, laboratory results, and free text of clinical notes	Automated surveillance for influenza should integrate information from prescriptions and free text clinical notes. Case detection with emergency medical records focusing on influenza-like cases with fever can reduce delay and workload to detect influenza epidemics
C. I. Kiefe(BTI 02-092)	The VHA medical system can play an essential role following a biological terrorist attack or infectious outbreak due to its extensive record in disaster preparedness	To develop and test web-based teaching modules to increase VHA clinicians’ knowledge about biological warfare agents	Web-based educational intervention was tested at 15 VHA facilities via a randomized controlled trial with 332 participants	The VHA program demonstrated higher anthrax, but not smallpox, post- training provider knowledge than the information offered on the CDC’s website
M. Sano(BTI 02-233)	Limited efforts to prepare general public for a bioterrorism incident have been conducted	To develop educational materials for veterans about bioterrorism; to provide coping mechanisms for getting though a bioterrorism incident; to evaluate methods for material delivery	A Veterans’ Survey on Bio-Terrorism (VSOB) (the initial and a follow up) was mailed to 2923 veterans	VSOB, the first instrument to evaluate veterans’ knowledge, attitudes, beliefs, anxiety and educational needs connected to bioterrorism, was developed
A. Dobalian(RRP 06-134)	Existing research within and outside of VHA does not sufficiently address health issues for mentally ill and/or frail veterans during evacuations	To understand evacuation and response in VHA nursing homes after Hurricanes Katrina and Rita	Data were collected via 13 semi-structured interviews with organizational representatives at 4 VHA medical centers and two representatives at the regional level	Administrators primarily relied on local resources, prior experience and local planning rather than on state and federal response systems in their response to the hurricanes. Despite significant difficulties during patient evacuation, VHA response was generally perceived as positive. Retaining staff and a viable organization during and after a disaster presented a difficulty. Respondents reported unaddressed preparedness needs even more than one year post-disaster
F. M. Weaver(RRP 06-135)	Individuals with spinal cord injuries and disorders are at particular risk during disasters due to impaired mobility and special needs, such as power wheelchairs and ventilator dependency	To use identified lessons learned from natural disasters that impacted veterans with spinal cord injuries and disorders (SCI&D) in developing a toolkit, which focuses on enhancing natural disaster preparedness for facilities caring for veterans with SCI&D	Thirty interviews were conducted (16 with providers and 14 with veterans with SCI&D). Most interviewees had experienced at least one weather-related natural disaster	Veterans with SCI were usually evacuated to unaffected areas or were admitted to SCI centers. Previous disaster experiences provided lessons to guide providers’ and veterans’ actions. Pre-established response plans served as useful starting points. Family and local agencies’ social support was essential for veterans to attain a sense of personal preparedness. The above information was used to develop tools for disaster preparedness.
B. Schmitt(IIR 02-080)	VHA is particularly vulnerable to a postal attack directed at government facilities. Thus, it has an interest in identifying the most advantageous response to small and large-scale bioterrorist events	To conduct a cost-effectiveness analysis comparing response strategies to a small and a large-scale anthrax attack	A decision analytic model was used to compare 3 basic response strategies to a small scale anthrax attack. The optimal response to a mass inhalation anthrax attack was evaluated. Outcomes included costs, Quality-Adjusted Life Years, and incremental cost-effectiveness	For the small-scale anthrax attack, the least costly strategy was administration of antibiotics post-attack; post-attack antibiotic and post-attack vaccination strategy was the most effective. Pre-attack vaccination was the least effective. Pre-attack vaccination was preferable to post-attack antibiotics alone when the probability of anthrax exposure was ≥16%. For the large-scale mass attack scenario, analysis is in progress

*Systematic Literature Review*

A systematic literature review was conducted to synthesize what is known about VHA emergency management research. Specifically, the review answered the following research questions: 1. What is the role of VHA in emergency management, including mitigation, preparedness, response, and recovery? 2. For each of the identified VHA emergency management activities, what recommendations (“lessons learned”) were made to improve the activity around mitigation, preparedness, response & recovery? 3. What veteran health needs have been identified as important in emergency management? Results of the review are presented elsewhere.^6^

**Achieving Consensus on Research Priorities**

The purpose of the VHA Comprehensive Emergency Management Program Evaluation and Research Conference was to bring together researchers and practitioners in a common forum to discuss and make recommendations regarding the direction of future VHA program evaluation and research on emergency management. Participant affiliations included VHA, various universities, CDC, the Department of Defense, NIH, and AHRQ, and other institutions.

After several context-setting presentations about VHA’s role in emergency management, attendees participated in one of five workgroups: Behavioral Health (e.g., mental health; substance use/abuse; psychological first aid; the “worried well”); Workforce (e.g., education/training of personnel; VHA’s Disaster Emergency Medical Personnel System (DEMPS); competing family concerns); Communication and Information Flow (e.g., decision-making; inter-organizational collaboration; risk communication); Sustainability and Resilience (e.g., quality improvement; community resilience); and Systems Capabilities (e.g., broad health systems issues such as evacuation, pandemic influenza; methodological considerations when conducting research in this field; inter-organizational collaboration).

## VHA’s Emergency Management Research Agenda

**Behavioral Health**

The Behavioral Health workgroup (e.g., mental health; substance use/abuse; psychological first aid; the “worried well”) was tasked with identifying and prioritizing VHA emergency management research issues related to the mental health needs of individuals impacted by a disaster or mass casualty event. While the conversation did include some discussion of VHA workforce needs (e.g., training and psychological support) as well as the mental health impacts of disasters on VHA and VHA’s role in providing care for the larger community, most of the workgroup’s discussion focused on preventing and treating post-disaster development or exacerbation of behavioral health problems in the veteran population. Because VHA provides ongoing medical and support services for many veterans with psychological and substance use disorders, addressing the impact of large scale emergencies and disasters on behavioral health needs was considered to be of particular significance to VHA.

Priorities

1. Preventing and treating post-disaster development or exacerbation of behavioral health problems among veterans^7^^,^^8^
2. Identification and evaluation of existing post-disaster mental health interventions among veterans
3. Examining the impact of large-scale emergencies and disasters on behavioral health needs of veterans

Key Questions/Research Topics

1. How can VA mitigate the impact of a disaster or mass casualty event on the physical, psychological, and social functioning of veterans with pre-existing or emergency behavioral health issues?
2. How does VA assure adequate and appropriate post-disaster access to quality emergency behavioral health services, both for veterans receiving services from VA and for veterans seeking VA health services for the first time? Examples of specific questions within this broad area of inquiry included:
  1. What systems are in place to assure access and continuity of care for current VA health care users who are displaced or impacted by an event?
  2. What administrative processes are necessary for a veteran who is new to VA to receive services and is there a need to expedite or modify these processes to provide emergency behavioral health services?
  3. What type of behavioral health services will be most needed post-disaster?
  4. Who is likely to seek post-disaster behavioral health services through VA?
  5. How can VA identify veterans who may need post-disaster behavioral health services, but are not likely to seek out or access the services?
3. What post-disaster mental health interventions are currently used by VA? Who provides the interventions and how are the providers trained? How, if at all, are the interventions evaluated? What is the association between these early intervention strategies and long-term mental health outcomes?
4. Assess the psychosocial consequences of disasters and emergencies for the “worried well,” and develop evidence-based strategies to minimize those consequences.
5. How, once identified, do we build “best practices” for addressing the behavioral health impacts of disasters into VA’s emergency management system?

**Workforce**

The Workforce workgroup (e.g., education/training of personnel; Disaster Emergency Medical Personnel System (DEMPS)^9^; competing family concerns) focused on issues regarding: designing and evaluating effective education and training strategies for health care personnel, establishing competency guidelines, effectively engaging health care providers in decision making related to emergencies, DEMPS teams, and how to address employees’ competing concerns for the safety of their own family members. There was a general consensus that a fair amount of funding has been dedicated to training and education, but that rigorous research about the effectiveness of training and education programs is lacking. Furthermore, future effectiveness research should differentiate demonstrating competencies of individuals from system capacity, which is dependent on infrastructure. The workgroup recognized the importance of VHA’s work with various federal partners, and that the manner in which it interacts with other federal agencies is a critical area of research regarding workforce issues.

Priorities

1. Rigorous research about the effectiveness of training and education programs is lacking
2. Research should differentiate between demonstrating competencies of individuals from systems capacity, which is dependent on infrastructure

Key Questions/Research Topics

1. Evaluation
  1. What evaluation methods are currently being used to assess workforce training outcomes?
  2. How are exercises being used to assess workforce competency?
  3. Develop recommendations for optimizing the learning potential for attendees who participate in emergency management exercises.
  4. How does prior training of staff affect performance?
  5. Develop recommendations for the most effective methods of learning their roles and responsibilities before, during and after disasters for each of the various job groups within VHA’s emergency management competency framework (all employees; health system leaders; patient care providers; clinical support; facilities and engineering; law enforcement; and, emergency program managers).
2. Leadership Skill Development
  1. To what extent is the leadership at VA facilities able to apply FEMA 100-800 standardized training to its facility?
  2. Is further training necessary and in what areas?
  3. How do VA facility leaders’ expectations for preparedness differ across staff?
  4. What questions does VA leadership have about workforce and emergency management and preparedness (needs assessment)?
3. Optimally Utilizing Existing Data
  1. How can the Comprehensive Emergency Management Program (CEMP) hospital readiness data that was collected by VA in 2008-10 be used to answer questions about workforce and emergency preparedness?^10^^,^^11^
  2. What are the mutable and immutable characteristics of high-performing systems identified by VA hospital readiness evaluations in 2008-10?
4. Relationship between Local and National Preparedness
  1. What locally-provided training predisposes individuals to be more effective during national deployments?

**Communication & Information Flow**

The Communication and Information Flow workgroup (e.g., decision-making; inter-organizational collaboration; risk communication) focused on a wide array of topics related to emergency management planning issues within VHA, specifically crisis communication, risk communication, communication tools, and community collaboration applicable to the overall healthcare system, veterans, staff, and the community. The group expressed the potential concern that VHA’s organizational culture may be overly driven by protocols and standards, and questioned whether communication could effectively be structured to make and disseminate clinical and strategic decisions to veterans, staff, and local communities in an effective and time-efficient manner given these concerns. The group indicated that it would be valuable to identify triggers that lead to the effective dissemination of information from VHA to the public, and wondered how those mechanisms would be altered by a public health emergency. In this regard, the workgroup noted that VHA could also draw on the expertise of its federal and other partners.

Priorities

1. Decision-making process
2. Communication with external audience
3. Crisis communication strategies and management
4. Internal communication, decision-making and information management
5. Communication tools and techniques
6. Media
7. Culture and culture change

Key Questions/Research Topics

1. How effective is decentralized decision-making for the veteran population and VA community?
2. How well does VA communicate with veterans (particularly for certain groups of veterans such as the homeless, veterans in the community) and the public?
3. Determine the most effective strategies for communicating with at-risk veterans before, during, and after impacts from hazards, and provide recommendations on how these strategies may change based on the nature of the hazard or the particular group of at-risk veterans.
4. How well integrated is crisis communication integrated in VA preparedness and response activities? How effective is it?
5. Is communication flow from decision-making to clinicians and staff adequate?
6. Determine the most effective strategies for communicating with employees after major disasters.
7. How does VA effectively monitor social media and respond?
8. How effectively is VA partnering and working with the media around emergency management?
9. How well does VA’s general employee culture adapt to the disaster and response culture?

**Sustainability and Resilience**

The Sustainability and Resilience workgroup (e.g., dual-use systems that may improve the quality of care delivered outside of a disaster situation as well as in the event of an emergency; community resilience) met to discuss the sustainability of resources for emergency preparedness and response, and was asked to consider areas in which to invest scarce resources; quality and cost; how to leverage existing systems or to establish “dual-use systems” that provide benefits both under non-emergent and emergent situations; challenges related to the ebb and flow of funding related to the recency and size of a domestic disaster; and the resilience of veterans and VHA, as well as community resilience in general. The workgroup stressed the importance of disaster research funding and recommended that such funding be increased as a prerequisite for a successful emergency management research agenda and its ongoing implementation.

Priorities

1. Emergency management visibility and capability building
2. Community integration
3. Supply chain limitations (especially pharmaceutical caches and hospital bed capacity)
4. Staff resilience and other resilience issues^9^^,^^12^
5. Special needs patients^13^^,^^14^^,^^15^^,^^16^^,^^17^

Key Questions/Research Topics

1. Who and what organizations constitute and are possible and probable partners of VA, especially during an event?
2. What tools must be developed to map out networking, communications, and cooperation opportunities with community’s healthcare providers and public health departments?
3. How can VA gauge the awareness of supply chain limitations?
4. What tools must be developed to identify the locations of national and VA suppliers?
5. How can VA compare actual versus theoretical hospital bed capacity?
6. What training should VA staff receive to lower mental health stress (especially during an event), maintain proficiency, and be adequately and appropriately cross-trained?
7. What role can VA play in leading community resilience efforts?
8. How can VA examine and test community resilience?
9. How to assess needed support systems to assure access to services and continuity of care for veterans who are displaced or otherwise impacted by a disaster or emergency?
10. What strategies and tactics should VA treatment facilities incorporate into their emergency management programs to be ready for the effects of convergence during and after community disasters?
11. How can VA coordinate emergency preparedness/management efforts within a broader community?
12. Identify the targets, frequency and nature of collaborations necessary for VA treatment facilities can use to establish effective mutual-aid relationships with community health care partners and public safety agencies.
13. How can VA map out networks/cooperation between VA and local healthcare providers?
14. Who is considered a special needs patient?
15. What are the most vulnerable populations of veterans?
16. Ascertain the percentage(s) and locations of veterans who fall into one or more socioeconomic or demographic categories for those who are considered at higher risk from the effects of hazards.
17. Develop recommendations to address the special needs of veterans in general, and specific vulnerable populations of veterans including veterans with cognitive or functional impairments (e.g., those with traumatic brain injuries or spinal cord injuries), homeless veterans, veterans living with HIV/AIDS, frail veterans in the community (e.g., those needing oxygen), and veterans with posttraumatic stress disorder (PTSD).
18. What is the backup plan to assist veterans with special needs during an event?

**Systems Capabilities**

The Systems Capabilities workgroup (e.g., broad health systems issues such as evacuation, pandemic influenza; methods; inter-organizational collaboration) discussed the broader healthcare system and population issues applicable to all healthcare systems, although it focused primarily on VHA-specific issues while considering both internal and external concerns. Using both experience with current practices, including a discussion of actual operational decisions made during the response by VHA and others to Hurricane Katrina, as well as an assessment of current gaps in the field’s understanding, this workgroup identified various research priorities. Much of the workgroup’s discussion concerned the potential for VHA to become a leader in developing evidence-based standards for emergency management. The workgroup noted that VHA’s facilities and other resources provide an invaluable “laboratory” to strengthen national emergency management research capabilities. Resources noted by the workgroup included the recognition that VHA facilities that provide care for the most complex inpatient cases are required to have academic resource centers in their facilities. In addition, VHA staff with a military background often have experience either training for or actually having responded to a disaster. Furthermore, VHA currently makes resources available to the local community, including pamphlets that describe how to respond to a local emergency, and plays an extensive role in national emergency response. Finally, in rural areas, VHA may be the sole federal presence in the community and is often relied upon as the primary source of federal distribution, care and support. Members of the workgroup who had been part of the Katrina response also discussed issues surrounding surge capabilities where healthcare workers from neighboring institutions were farmed out to distant facilities because their hospitals were closed.

Priorities

1. Evacuation and sheltering in place^18^^,^^19^^,^^20^^,^^21^
2. Develop “off-the-shelf” evaluation protocols and surveys for use in the immediate aftermath of an event
3. Developing common standards of practice, looking both nationally across VAs as well as within local community settings
4. Staffing variables to consider when responding to a multi-casualty incident since VAMCs are typically not acute trauma centers
5. Breakdown of communication within a community, in particular when regular communication lines are down
6. Assessing actual volunteer capabilities

Key Questions/Research Topics

1. Develop decision support tools that healthcare providers and officials would need in the event of various disasters and emergencies.
2. The development of criteria and algorithms for evacuating patients, to be determined pre-incident and to guide the process of how to make decisions effectively.
  1. Assess current hospital and nursing home evacuation procedures and develop recommendations to improve their effectiveness.
3. When is it safer for an institution to not evacuate, but rather focus on facility hardening, sufficient supply, and shelter/protection (i.e. shelter in place)?
4. Determine the level of investment necessary to retrofit VHA’s current building inventory (business occupancies) to the effects of wind, water, fire and ground-shaking.
5. Define, measure, and evaluate surge capacity within VHA.
  1. How to address surge capabilities, such as when personnel are sent to distant facilities because local facilities are closed.
6. How to best build interest in the importance of emergency management at all delivery levels (e.g., the CMOs).
7. How can the six major capabilities of the VHA hospital readiness data collected in 2008-10 be used to establish a framework for evaluation?
8. Review the current VHA Capabilities Assessment Program and recommend strategies for enhancing future evaluation strategies and methods.
9. Assess the current effectiveness and develop improvement strategies related to VHA’s health information technology in detecting, tracking, and providing real-time decision support to clinicians.
10. What lessons may be learned from the use of Federal Medical Stations (FMS) as a lab for post-incident patient care?
  1. The types of equipment used to support these facilities, such as having sufficient equipment to support obese patients.
  2. The FMS can also be used to examine the effects of altered standards of care in post-incident situations on healthcare professionals themselves.
11. Compare currently available methods for assessing risk, probability and vulnerability for VHA treatment facilities and develop recommendations for the most effective approach.
12. How to evaluate the effectiveness of current competencies required of VA employees? How effective are they in actual emergency responsiveness?
13. Develop evaluation strategies to support exercises and drills.
14. What equipment does a VA treatment facility need to effectively protect its employees and patients from the effects of an influenza pandemic?

## Building an Infrastructure for Fostering the Conduct of VHA Emergency Management Research

Based on the first three steps, the research team recommended a variety of measures to assure that there is adequate infrastructure within VHA to support the implementation of the research agenda. We recommended that a VHA agenda-setting process be reconvened within five years to assess progress on implementing the agenda and to establish new directions for subsequent years. The current research agenda was developed based on the best available data at the time. We anticipate that VHA’s investments in emergency management program evaluation and research will continue to yield rapid advances. This translates into a rapidly changing landscape, and a new set of knowledge and investigators who should be brought together to reappraise, re-energize, and recommit to the next phase of VHA emergency management evaluation and research. Although an updated agenda-setting conference has not been reconvened as of 2016, the initial conference was followed in subsequent years by annual meetings (Advancing and Redefining Communities for Emergency Management) that continue to bring together VA and non-VA researchers, practitioners, and policy-makers to share evidence-based practices and discuss the current state of emergency management research.

Some key differences exist between the VHA and other hospitals and healthcare facilities. For example, VHA has a well-integrated electronic medical system. Private facilities have begun to expand these capabilities in recent years. Electronic medical systems have advantages, but do require power to operate, and thus may require paper backups or other options during some disasters. In addition, VHA has a wide array of facilities that serve various populations, including residential facilities that serve Veterans with substance use disorders and various residential facilities for homeless Veterans.

In addition, it was recommended that efforts be made to increase the visibility of VHA’s emergency management research and its potential to serve as a laboratory for emergency management research for the Nation. Hospital systems often focus on healthcare-related issues at the expense of applying findings from the broader disaster-related literature.^22^^,^^23^^,^^24^ It was hoped that issues such as this could be explored within VHA for the benefit of both VHA and the nation. In particular, VHA should maintain and expand a searchable database of published articles and unpublished reports related to VHA emergency management program evaluation and research that would provide support to VHA researchers interested in VHA emergency management research opportunities and collaborations. This effort could lead to the establishment of a multi-component, web-based emergency management evaluation resource clearinghouse that would make emergency management research and evaluation resources more readily available and accessible to researchers and practitioners. Similarly, the establishment of a VHA Comprehensive Emergency Management Program Evaluation Center would enhance VHA’s mitigation, preparedness, response, and recovery activities in the event of emergencies and disasters. The Center’s goal should be to develop an evidence base by which VHA contributes to the development, evaluation and improvement of healthcare services and programs that (1) strengthen VHA’s CEMP, and (2) position VHA as a national leader in emergency preparedness and response. As a result of these recommendations, VHA established the Veterans Emergency Management Evaluation Center (VEMEC) in July 2010. VEMEC continues to this day.

## Conclusions

Using a systematic evidence base and consensus development process among stakeholders within and outside VA, we report on the first national VA comprehensive emergency management program evaluation and research agenda. VA provides a unique national laboratory for the conduct of high quality research that will improve VA’s and our Nation’s emergency medical and public health preparedness and the role of health delivery systems in that endeavor. To effectively foster the conduct and expansion of emergency management evaluation and research within VA, the consensus was that VA needs to build program evaluation capacity, increase the awareness and visibility of VA’s emergency management research, and build bridges to research partners at agencies and organizations with longstanding commitments to advancing emergency management research.

## Corresponding Author

Aram Dobalian: aram.dobalian@va.gov

## Competing Interests

The authors have no financial relationships or conflicts of interest to disclose.

## Data Availability

All relevant data are available from the figshare repository: https://dx.doi.org/10.6084/m9.figshare.3085807.
